# 3-Hydroxy-β-ionone Suppresses Breast Cancer Progression by Inducing Apoptosis and Blocking EMT Through the TGF-β/Smad Signaling Pathway

**DOI:** 10.3390/ijms26188771

**Published:** 2025-09-09

**Authors:** Pornsuda Sutana, Thitiya Luetragoon, Watunyoo Buakaew, Krai Daotak, Nungruthai Nilsri, Yordhathai Thongsri, Pachuen Potup, Catherine Léon, Kanchana Usuwanthim

**Affiliations:** 1 Cellular and Molecular Immunology Research Unit (CMIRU), Faculty of Allied Health Sciences, Naresuan University, Phitsanulok 65000, Thailand; pornsudas64@nu.ac.th (P.S.); nok.hong.yok49@gmail.com (T.L.); watunyoo@g.swu.ac.th (W.B.); kraid@nu.ac.th (K.D.); nungruthaini@nu.ac.th (N.N.); yordhathait@nu.ac.th (Y.T.); pachuenp@nu.ac.th (P.P.); 2 Department of Medical Technology, Faculty of Allied Health Sciences, Nakhonratchasima College, Nakhonratchasima 30000, Thailand; 3 Department of Microbiology, Faculty of Medicine, Srinakharinwirot University, Bangkok 10110, Thailand; 4 INSERM, UMR_S1255, Université de Strasbourg, Etablissement Français du Sang-GEST, 67000 Strasbourg, France; catherine.leon@efs.sante.fr

**Keywords:** 3-hydroxy-β-ionone, triple-negative breast cancer, *Moringa oleifera*, molecular docking, molecular dynamic simulation

## Abstract

Triple-negative breast cancer (TNBC) is the most aggressive breast cancer subtype, with limited treatment options and a poor prognosis. Epithelial-to-mesenchymal transition (EMT) plays a critical role in promoting TNBC metastasis. The natural bioactive substance 3-hydroxy-β-ionone (3-HBI), which has been studied in other cancer types, has not yet been examined in TNBC. This study investigates its potential mechanisms in TNBC cells through a combination of computational and experimental approaches, focusing on apoptosis induction and EMT inhibition. Molecular docking and molecular dynamics simulations demonstrated strong and stable binding of 3-HBI to key apoptosis-related proteins (Bcl-2, Bax, caspase-3) and EMT regulators (Smad2, Smad3). In vitro, 3-HBI significantly reduced cell viability in MDA-MB-231, T47D, and MCF7 cells, with IC_50_ values of 388.40, 185.50, and 113.40 µg/mL, respectively. Moreover, 3-HBI suppressed clonogenic potential, induced apoptosis, and inhibited both migration and invasion. Molecular analysis showed increased expression of Bax, caspase-3, and E-cadherin, and reduced levels of Bcl-2, Smad2, Smad3, and N-cadherin. These findings provide the first mechanistic evidence that 3-HBI exerts anti-TNBC effects by promoting apoptosis and suppressing EMT, highlighting its potential as a promising therapeutic candidate for TNBC treatment.

## 1. Introduction

Breast cancer is characterized by the uncontrolled proliferation of mammary epithelial cells and is the most commonly diagnosed cancer in women worldwide, with high incidence and mortality rates [1,2]. Triple-negative breast cancer (TNBC) is considered an aggressive subtype, accounting for 15–20% of all breast cancer cases. It is characterized by the absence of estrogen receptors (ER), progesterone receptors (PR), and human epidermal growth factor receptor 2 (HER2), rendering it unresponsive to current hormone or HER2-targeted therapies [3]. Chemotherapy remains the mainstay treatment for TNBC; however, it is often associated with adverse effects such as lymphedema, neuropathy, and systemic toxicity. In addition, TNBC is aggressive, with early visceral metastases, high rates of recurrence and medication resistance, and a worse prognosis than other subtypes of breast cancer [4,5].

The epithelial-to-mesenchymal transition (EMT) is a critical process that facilitates tumor progression, invasion, and chemoresistance. In breast cancer, EMT is largely regulated by the transforming growth factor beta (TGF-β)/mothers against decapentaplegic homolog (Smad) signaling pathway, in which phosphorylated Smad2 and Smad3 form a complex with Smad4 and translocate into the nucleus to regulate EMT-related genes such as N-cadherin and Vimentin [6,7,8,9]. Increased TGF-β signaling is strongly associated with tumor grade, metastasis, and poor clinical outcomes in TNBC [9,10]. Moreover, TGF-β signaling can modulate cancer cell stemness and suppress apoptosis, further contributing to therapeutic resistance [5,6,7,8,9,10]. Consequently, inhibiting the EMT process by suppressing the TGF-β/Smad signaling pathway plays a critical role and represents a promising therapeutic approach for TNBC treatment.

Natural compounds, including alkaloids, flavonoids, terpenoids, and phenylpropanoids, have emerged as promising anti-cancer agents due to their biological activity and low toxicity [11,12,13]. 3-hydroxy-β-ionone (3-HBI) found in *Moringa oleifera* Lam. (MO) [14] and the Bangladeshi rice cultivar Kartikshail [15] and *Rhynchostegium pallidifolium* [16] have demonstrated anti-inflammatory [14] and anti-proliferative activities of head and neck cancer [17], hepatocellular carcinoma [18], and lung cancer [19]. However, the mechanistic role of 3-HBI in TNBC, particularly its effects on apoptosis and EMT through the TGF-β/Smad pathway, has not been studied.

In this study, we used both in vitro and in silico methods to examine the anti-cancer properties of 3-HBI found in MO extract in MDA-MB-231, a TNBC cells. We assessed its impact on invasion and migration, apoptosis induction, and cytotoxicity. Key target proteins in the apoptosis and EMT pathways were identified using molecular docking and molecular dynamics simulations, and these were then experimentally validated using Western blot and RT-qPCR. Here, we show that 3-HBI suppresses breast cancer by causing apoptosis and blocking invasion and migration. Additionally, the study suggests that 3-HBI may target Smad2 and Smad3 in the TGF-β/Smad pathway, Bcl-2–associated X (Bax), B-cell lymphoma 2 (Bcl-2), and caspase-3 in the apoptosis pathway in order against TNBC.

## 2. Results

### 2.1. The Cytotoxicity of 3-Hydroxy-β-ionone (3-HBI)-Identified from Moringa oleifera Lam. (MO) Extract on Breast Cancer Cell Lines

**Figure 1:**
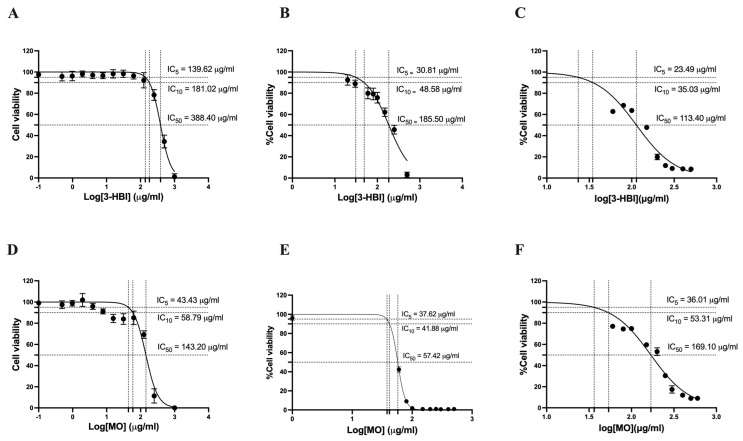
Effect of 3-HBI and MO extract on the cell viability of breast cancer cell lines and their IC_5_, IC_10_, and IC_50_. (**A**) 3-HBI on MDA-MB-231 (**B**) 3-HBI on T47D (**C**) 3-HBI on MCF7 (**D**) MO extract on MDA-MB-231 (**E**) MO extract on T47D (**F**) MO extract on MCF7. IC: inhibitory concentration; 3-HBI: 3-hydroxy-β-ionone; MO: *Moringa oleifera* Lam.

To determine the cytotoxic potential of 3-HBI and MO extract, MTT assays were conducted on TNBC cells. 3-HBI exhibited dose-dependent cytotoxicity in MDA-MB-231, as the TNBC cell line. The five percent inhibitory concentration (IC_5_), the ten percent inhibitory concentration (IC_10_), and the half-maximal inhibitory concentration (IC_50_) values of 3-HBI on the MDA-MB-231 were 139.62 μg/mL, 181.02 μg/mL, and 388.40 μg/mL, respectively (Figure 1A). The corresponding IC_5_, IC_10_, and IC_50_ values for MO extract on MDA-MB-231 were 43.43 μg/mL, 58.79 μg/mL, and 143.20 μg/mL, respectively (Figure 1D). We also demonstrated the anti-breast cancer properties of 3-HBI in other breast cancer subtypes that are known to exhibit greater drug sensitivity than TNBC. Specifically, ER-positive and PR-positive breast cancer cells (T47D) and ER-positive breast cancer cells (MCF7) were tested using concentrations slightly above the IC_50_ value determined for MDA-MB-231 cells. 3-HBI exhibited dose-dependent cytotoxicity in both T47D and MCF7 cells, similar to that observed in MDA-MB-231 cells.

IC_5_, IC_10_, and IC_50_ values for 3-HBI on T47D were 30.81 μg/mL, 48.58 μg/mL, and 185.50 μg/mL, respectively (Figure 1B). IC_5_, IC_10_, and IC_50_ values for MO extract on T47D were 37.62 μg/mL, 41.88 μg/mL, and 57.42 μg/mL, respectively (Figure 1E). The IC_5_, IC_10_, and IC_50_ values for 3-HBI extract on MCF7 were 23.49 μg/mL, 35.03 μg/mL, and 113.40 μg/mL, respectively (Figure 1C). IC_5_, IC_10_, and IC_50_ values for MO extract on MCF7 were 36.01 μg/mL, 53.31 μg/mL, and 169.10 μg/mL, respectively (Figure 1F).

### 2.2. The Cytotoxicity of 3-HBI on the Fibroblast Cell Line

**Figure 2:**
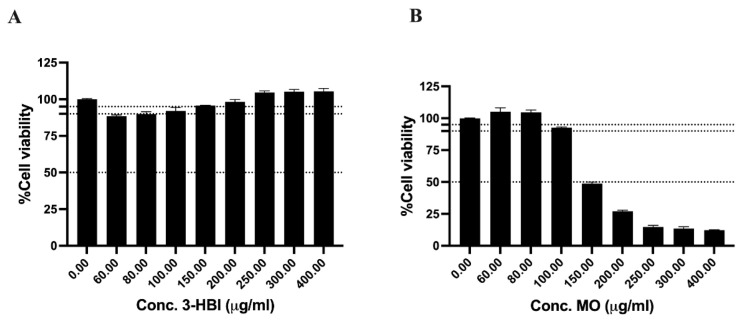
Cell viability of the CCD-1123Sk cell line. (**A**) 3-HBI treatment and (**B**) MO extract treatment. 3-HBI: 3-hydroxy-β-ionone; MO: *Moringa oleifera* Lam.

To evaluate the potential of 3-HBI as a future therapeutic agent against triple-negative breast cancer (TNBC), we investigated its effects on normal, proliferating human cells. Specifically, we employed the human dermal fibroblast cell line CCD-1123Sk to model the cytotoxic response in non-cancerous cells, recognizing that natural compounds often exhibit limited specificity and may adversely affect rapidly dividing normal cells [20,21]. The results showed that MO reduced fibroblast viability in a dose-dependent manner (Figure 2B). In contrast, treatment with 3-HBI led to an increase in fibroblast cell viability (Figure 2A). Notably, the IC_50_ values observed for all tested breast cancer cell lines (Figure 1) indicate that these doses were non-toxic to normal fibroblast cells (Figure 2). These results suggest that both 3-HBI and MO are non-toxic to human fibroblasts, with 3-HBI potentially offering a more favorable safety profile.

We selected concentrations of 3-HBI at 60, 80, and 150 μg/mL, and MO at 60, 80, and 100 μg/mL to investigate the effects of 3-HBI-derived MO on key hallmarks of TNBC. All concentrations used were below the IC_50_ values for TNBC cell lines and demonstrated safety in normal fibroblast cells. Additionally, doxorubicin was included as a positive drug control in all experiments. Doxorubicin, a commonly used chemotherapeutic agent, functions as a topoisomerase II inhibitor, thereby disrupting DNA replication and inducing cell death [22]. These parameters establish a foundation for future studies evaluating the therapeutic potential of 3-HBI in TNBC treatment.

### 2.3. 3-HBI Inhibited TNBC Colony Formation

To assess anti-TNBC activity, we investigated key cancer hallmarks: proliferation, apoptosis resistance, and metastasis. We performed 3-HBI on TNBC proliferation by using colony formation. The colony formation assay is an in vitro cell survival assay based on the ability of a single cell to grow into a colony.

**Figure 3:**
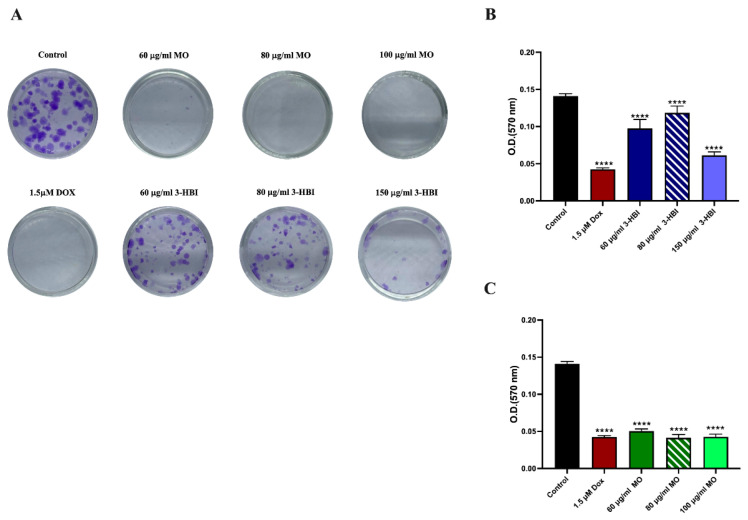
Colony formation assay of the MDA-MB-231 cell line was performed in 12-well plates, with cells stained using 0.5% crystal violet. (**A**) Colony formation in control groups, 3-HBI groups, and MO extract groups after 24 h treatment. (**B**) O.D. at 570 nm in the 3-HBI treatment group. (**C**) O.D. at 570 nm in the MO treatment group. Data are presented as means ± SEM. **** *p* ≤ 0.0001, compared to control. Control: untreated MDA-MB-231; 3-HBI: 3-hydroxy-β-ionone; MO: *Moringa oleifera* Lam.

Colony formation assays revealed a substantial decrease in the number of colonies in treated TNBC cells (Figure 3A). When dissolved, crystal violet was measured for absorbance at 570 nm, and 3-HBI treatment groups significantly reduced colony formation, similar to doxorubicin when compared to the control (Figure 3B). Additionally, MO reduced the colony formation of MDA-MB-231 (Figure 3A,C).

### 2.4. The Role of 3-HBI on Breast Cancer Cell Apoptosis

Apoptosis targeting demonstrates effectiveness against various types of cancer [23]. First, we hypothesized that 3-HBI interacts with apoptosis-related proteins and tested the stability of the resulting complexes using molecular docking and molecular dynamics simulations. Next, apoptosis in breast cancer cells was assessed using Annexin V/7-AAD staining. Finally, we confirmed the involvement of genes and proteins related to the molecular docking and molecular dynamics simulation results through reverse transcription-quantitative real-time PCR (RT-qPCR) and Western blot analysis.

#### 2.4.1. In Silico Binding to Apoptotic Regulators

**Figure 4:**
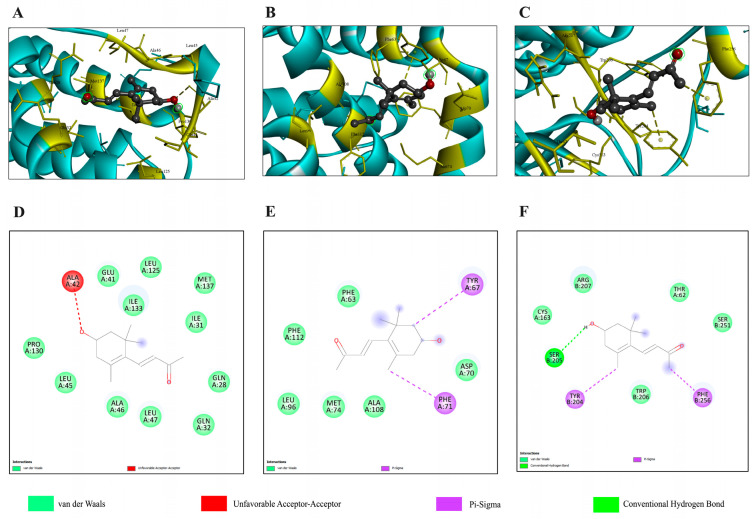
Docking interactions of 3-HBI with Bax, Bcl-2, and caspase-3. Three-dimensional arrangement of 3-HBI interacting with (**A**) Bax, (**B**) Bcl-2, and (**C**) caspase-3. Two-dimensional visualizations of 3-HBI interacting with (**D**) Bax, (**E**) Bcl-2, and (**F**) caspase-3. Bax: Bcl-2–associated X; Bcl-2: B-cell lymphoma 2.

The interactions between 3-HBI and each apoptosis pathway-related protein were analyzed using AutoDock Vina. The analysis revealed that the proteins with the highest probability of being target proteins are Bax, Bcl-2, and caspase 3, with ΔG values of −6.5, −5.9, and −5.4 kcal/mol, respectively, as shown in Table 1. Figure 4 provides detailed 2D and 3D visualizations of the predicted binding poses and specific chemical interactions between 3-HBI and Bax (Figure 4A,C), Bcl-2 (Figure 4B,E), and caspase 3 (Figure 4C,F).

**Figure 5:**
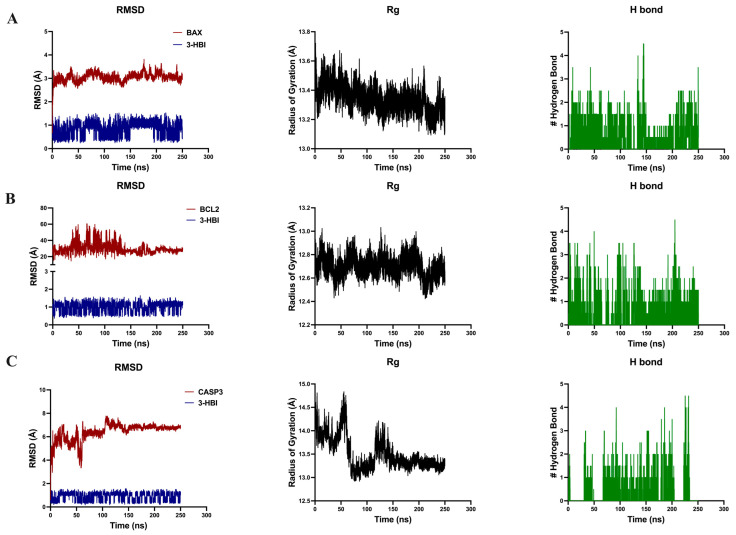
Molecular dynamics simulations of 3-HBI with Bax, Bcl-2, and caspase-3. It presents root mean square deviation (RMSD) plots, radius of gyration (Rg) plots, and hydrogen bond counts for (**A**) Bax, (**B**) Bcl-2, and (**C**) caspase-3. Bax: Bcl-2–associated X; Bcl-2: B-cell lymphoma 2.

Furthermore, we validated the stability of the formed complexes over 250 ns using molecular dynamic simulation. The root mean square deviation (RMSD) indicated that 3-HBI is relatively stable, with values below 2 Å over a time scale of 250 ns for three proteins. Specifically, Bax-3-HBI remained relatively stable within 2–4 Å over a time scale of 250 ns (Figure 5A), Bcl-2-3-HBI exhibited stability within 20–60 Å during the same period (Figure 5B), and caspase-3-3-HBI stabilized within 6–8 Å after 150 ns (Figure 5C). To assess the compactness of the complexes, we analyzed the radius of gyration (Rg). The Rg of Bax-3-HBI remained constant at 13.4 Å throughout the simulation (Figure 5A), while Bcl-2-3-HBI maintained a stable Rg of 12.7 Å over 250 ns (Figure 5B). Caspase-3-3-HBI, however, stabilized at 13.3 Å after 150 ns (Figure 5C). Hydrogen bond analysis revealed a higher number of hydrogen bonds in Bax-3-HBI compared to Bcl-2-3-HBI and caspase-3-3-HBI (Figure 5A–C), further supporting Bax-3-HBI as the most stable interaction. Nonetheless, experimental binding assays are required to validate these computational findings.

#### 2.4.2. 3-HBI-Induced Breast Cancer Cell Apoptosis

**Figure 6:**
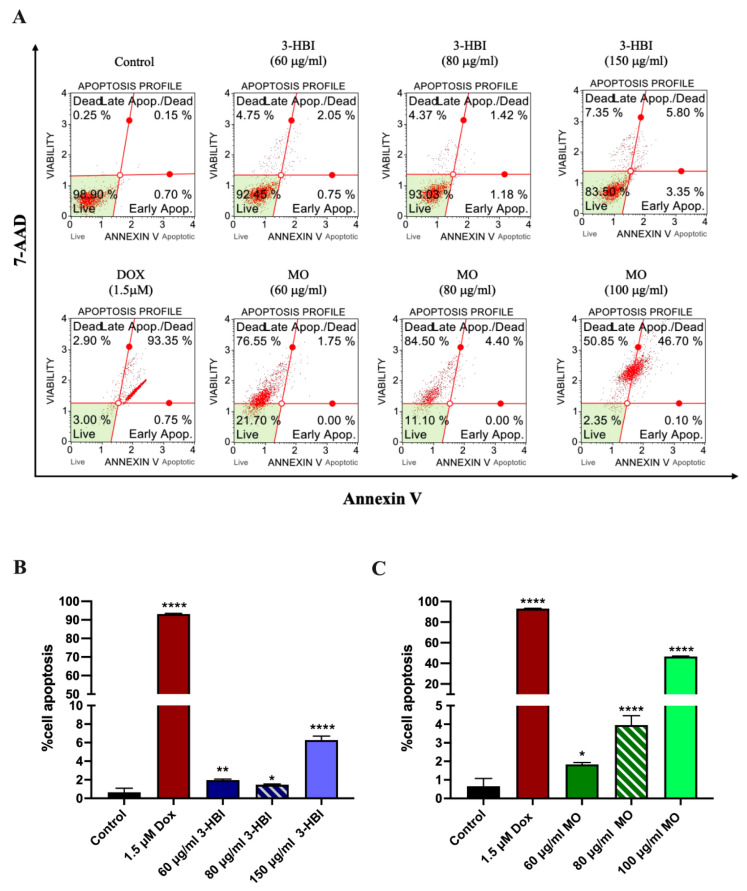
Apoptosis in the MDA-MB-231 cell line was analyzed using a Muse cell analyzer. (**A**) Apoptosis profiles of MDA-MB-23 cells. Bar graphs showing apoptosis in MDA-MB-231 cells for (**B**) 3-HBI treatment groups and (**C**) MO treatment groups. Data are presented as means ± SEM. * *p* ≤ 0.05, ** *p* ≤ 0.01, and **** *p* ≤ 0.0001 compared to control. Control: untreated MDA-MB-231; 3-HBI: 3-hydroxy-β-ionone; MO: *Moringa oleifera* Lam.

To evaluate 3-HBI-induced breast cancer apoptosis, after a 24 h treatment with MO extract and 3-HBI, the cells were assessed using Annexin V/7-AAD. The result showed that MDA-MB-231 cell apoptosis was significantly increased in a concentration-dependent manner with 3-HBI compared to the control (Figure 6A,B). Similar results were observed in MDA-MB-231 cells treated with MO extract and doxorubicin (Figure 6A,C). Moreover, we also demonstrated that 3-HBI can induce apoptosis in another breast cancer subtype by T47D. It was also shown that 3-HBI-derived MO extract induces apoptosis in T47D cells (Figure S1).

#### 2.4.3. The Effect of 3-HBI on Genes and Proteins in the Apoptosis Pathway

**Figure 7:**
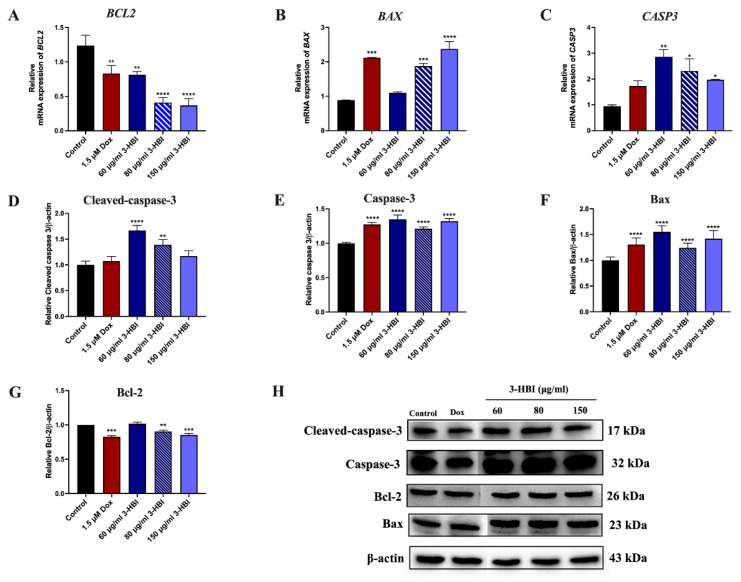
Effect of 3-HBI on apoptotic gene and protein expression. The expression of (**A**) *BCL2*, (**B**) *BAX*, and (**C**) *CASP3* genes in 3-HBI treatment groups. The bar graph represents apoptosis protein expression level of (**D**) cleaved-caspase-3, (**E**) caspase-3, (**F**) Bax, and (**G**) Bcl-2. (**H**) The picture of apoptosis protein expressions. Data are presented as means ± SEM. * *p* ≤ 0.05, ** *p* ≤ 0.01, *** *p* ≤ 0.001, and **** *p* ≤ 0.0001 compared to control. Control: untreated MDA-MB-231; 3-HBI: 3-hydroxy-β-ionone; *BAX* or Bax: Bcl-2–associated X; *BCL2* or Bcl-2: B-cell lymphoma 2; *CASP3*: caspase-3.

Molecular docking and molecular dynamics simulation-related key genes and proteins involved in apoptosis were validated by RT-qPCR and Western blotting analyses. RT-qPCR showed 3-HBI upregulation of Bax and caspase-3, and downregulation of Bcl-2 at the mRNA level (Figure 7A–C). Western blot analysis confirmed increased protein expression of Bax, caspase-3, and cleaved caspase-3, with a concurrent decrease in Bcl-2 (Figure 7D–H). These findings were consistent with the computational predictions, indicating apoptosis activation through the intrinsic pathway. This demonstrates the same outcome as the groups treated with MO extract (Figure S2).

### 2.5. 3-HBI Inhibits TNBC Metastasis

The TGF-β/Smad pathway is one of the key pathways involved in EMT [9,10], which plays a critical role in cancer metastasis. EMT is characterized by increased migration and the secretion of matrix metalloproteinases (MMPs) to degrade the extracellular matrix (ECM), enabling cancer cells to invade other sites for cancer metastasis [24,25]. Therefore, we predicted the interactions of 3-HBI with TGF-β/Smad pathway-related proteins using molecular docking and validated the stability of 3-HBI-protein complexes through molecular dynamics simulation. Then, to evaluate the effect of 3-HBI on TNBC metastasis, migration, and invasion assays were conducted. Moreover, Western blot and RT-qPCR assays were used to validate their interaction related to molecular docking and molecular dynamics simulation results.

#### 2.5.1. Molecular Docking and Molecular Dynamics Simulation with the TGF-β/Smad Pathway

**Figure 8:**
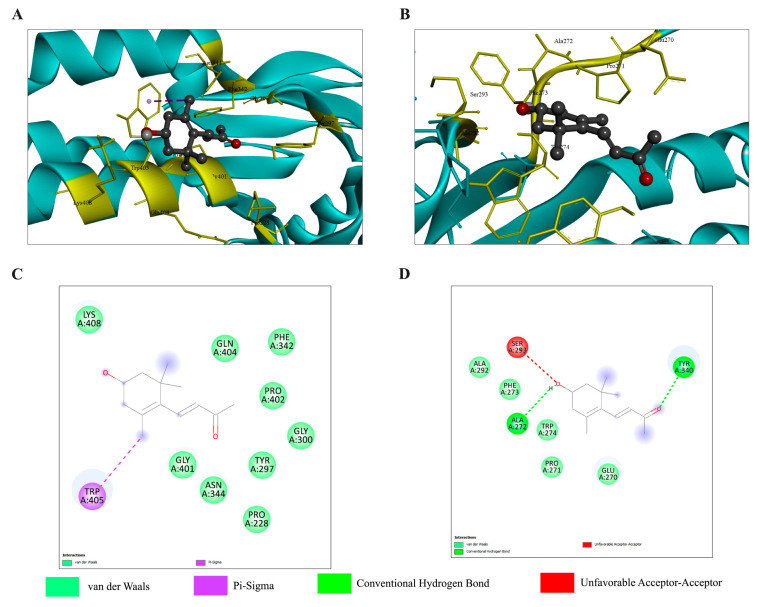
Docking interactions of 3-HBI with Smad2 and Smad3 in TGF-β/Smad pathway. Three-dimensional interaction of (**A**) Smad3, and (**B**) Smad2. Two-dimensional interaction of (**C**) Smad3 and (**D**) Smad2. TGF-β: transforming growth factor beta; Smad: suppressor of mothers against decapentaplegic; Smad2: suppressor of mothers against decapentaplegic 2; Smad3: suppressor of mothers against decapentaplegic 3.

To predict 3-HBI interactions with each TGF-β/Smad pathway-related protein involved in cancer metastasis, the proteins were analyzed using AutoDock Vina. It was found that the proteins with the highest probability of being target proteins are Smad3 and Smad2, with ΔG of −5.4 and −5.1 kcal/mol, respectively, as shown in Table 2. Figure 8 provides detailed 2D and 3D visualizations of the predicted binding poses and specific chemical interactions between 3-HBI with Smad3 and Smad2.

**Figure 9:**
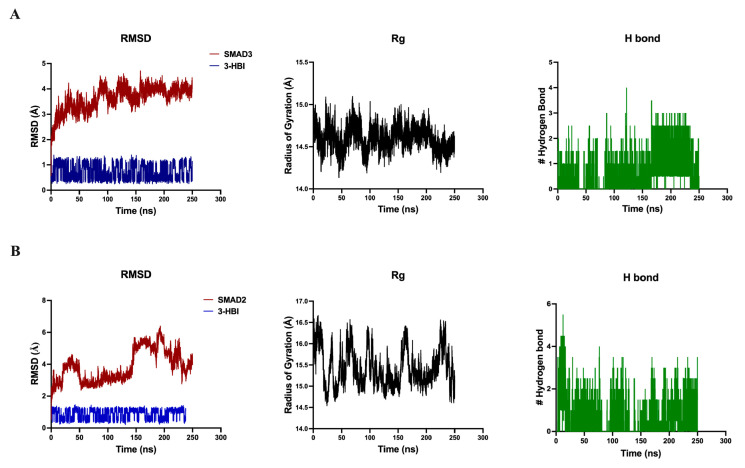
Molecular dynamics simulations of 3-HBI with Smad3 and Smad2 are presented, including root mean square deviation (RMSD) plots, radius of gyration (Rg) plots, and hydrogen bond counts for (**A**) Smad3 and (**B**) Smad2. Smad2: suppressor of mothers against decapentaplegic 2; Smad3: suppressor of mothers against decapentaplegic 3.

Further, the Smad3-3-HBI and Smad2-3-HBI constant complexes were verified by molecular dynamics simulation. In both complexes, 3-HBI remains relatively RMSD stable, with values below 2 Å over a 250 ns timescale. The RMSD of the Smad3-3-HBI complex remains consistent at 3–4 Å (Figure 9A), while the Smad2-3-HBI complex is around 3 Å in the period after 50 ns to before 150 ns. The Rg value of Smad3 (Figure 9A) is more constant than that of Smad2 (Figure 9B), indicating that the conformation of Smad3 protein is more stable than Smad2 and is less relaxed than Smad2. Hydrogen-bound analysis of both Smad3-3-HBI and Smad2-3-HBI are approximately the same number. Thus, the Smad3-3-HBI complex is the most stable interaction. However, experimental binding assays are needed to confirm the validity of these computational findings.

#### 2.5.2. 3-HBI Inhibited TNBC Migration and Invasion

To evaluate the effect of 3-HBI on EMT and metastatic potential in TNBC cells, we conducted wound healing and Transwell invasion assays. EMT is characterized by the transition of epithelial cells into mesenchymal-like cells, which enhances their migratory and invasive capacities. The wound healing assay was used to assess cell migration, reflecting the increased motility associated with EMT. In parallel, the Transwell invasion assay evaluated the ability of cells to degrade and ECM, a process facilitated by MMPs secreted during EMT [25]. Together, these assays provide functional insights into the role of 3-HBI in modulating EMT-related behaviors in TNBC cells.

**Figure 10:**
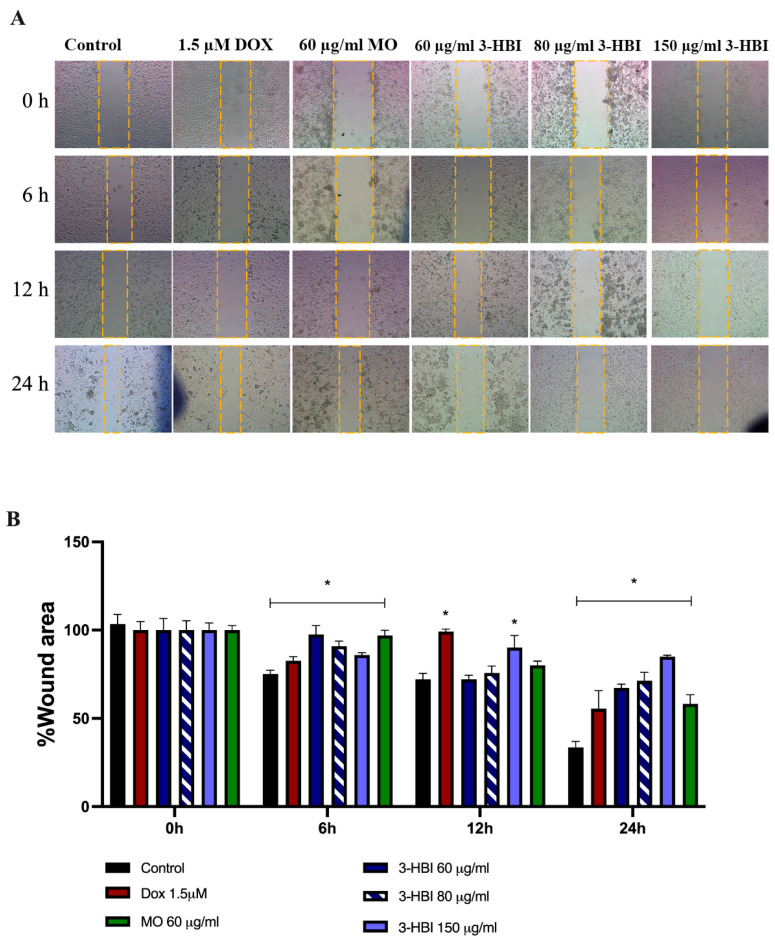
Migration of the MDA-MB-231 cell line. (**A**) Wound areas in untreated control groups, doxorubicin-treated groups, MO extract-treated groups, and 3-HBI-treated groups were observed at 0, 6, 12, and 24 h. (**B**) Bar graphs representing the percentage of the wound area. Data are presented as means ± SEM. * *p* ≤ 0.05 compared to control. Control: untreated MDA-MB-231; 3-HBI: 3-hydroxy-β-ionone; MO: *Moringa oleifera* Lam.

Wound healing demonstrated that 3-HBI, derived from MO, significantly suppressed TNBC cell motility. The wound area was assessed at 0, 6, 12, and 24 h. Compared to the untreated control, cells treated with MO extract or 3-HBI exhibited significantly delayed wound closure, with larger wound areas persisting throughout the observation period (Figure 10A). Quantitative analysis confirmed that both treatments reduced TNBC cell migration in a concentration-dependent manner (Figure 10B). Comparable inhibitory effects were observed in MDA-MB-231 cells treated with doxorubicin, serving as a positive control.

**Figure 11:**
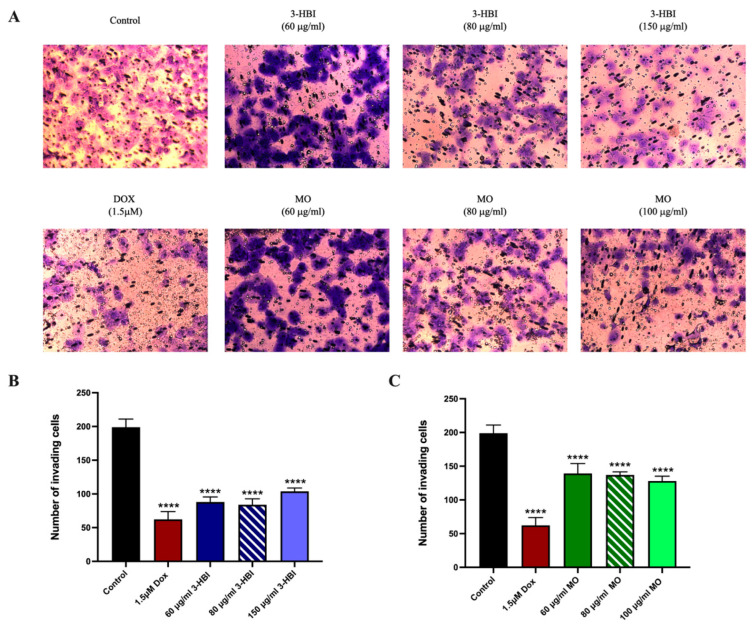
The invasion of the MDA-MB-231 cell line. (**A**) The invading cells in untreated control groups, doxorubicin-treated groups, MO extract-treated groups, and 3-HBI-treated groups after 24 h. (**B**) Bar graphs of 3-HBI. (**C**) Bar graphs of MO. Data are presented as means ± SEM. **** *p* ≤ 0.0001 compared to control. Control: untreated MDA-MB-231; 3-HBI: 3-hydroxy-β-ionone; MO: *Moringa oleifera* Lam.

An invasion assay was conducted to evaluate the impact of 3-HBI-derived MO extract on the invasion ability of TNBC. The findings demonstrated that, in comparison to the control, the invasion of cells was significantly reduced in the MO extract and 3-HBI groups as well as in the doxorubicin group (Figure 11).

#### 2.5.3. The Effect of 3-HBI on Genes and Proteins in the TGF-β/Smad Pathway

**Figure 12:**
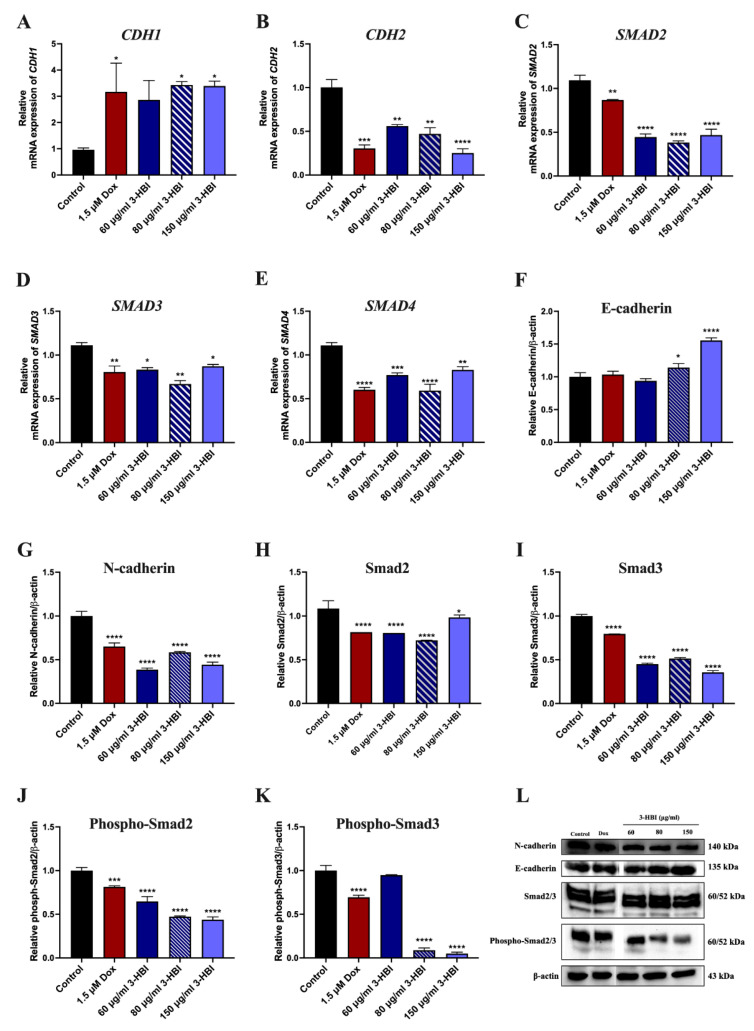
Effect of 3-HBI on TGF-β/Smad pathway-related gene and protein expression. The expression of (**A**) *CDH1*, (**B**) *CDH2*, (**C**) *SMAD2*, (**D**) *SMAD3*, and (**E**) *SMAD4* genes in 3-HBI treatment groups. Bar graphs of (**F**) E-cadherin, (**G**) N-cadherin, (**H**) Smad2, (**I**) Smad3, (**J**) phospho-smad2, and (**K**) phospho-smad3 proteins. (**L**) N-cadherin, E-cadherin, Smad2/3, and phospho-Smad2/3 expression. Data are presented as means ± SEM. * *p* ≤ 0.05, ** *p* ≤ 0.01, *** *p* ≤ 0.001, and **** *p* ≤ 0.0001 compared to control. Control: untreated MDA-MB-231; 3-HBI: 3-hydroxy-β-ionone. TGF-β: transforming growth factor beta; Smad: suppressor of mothers against decapentaplegic; *CDH1*: E-cadherin; *CDH2*: N-cadherin; *SMAD2* or Smad2: suppressor of mothers against decapentaplegic 2; *SMAD3* or Smad3: suppressor of mothers against decapentaplegic 3; *SMAD4*: suppressor of mothers against decapentaplegic 4.

To confirm that 3-HBI inhibits the TGF-β/Smad signaling pathway, RT-qPCR and Western blot analyses revealed that 3-HBI and MO downregulated *SMAD2*, *SMAD3*, *SMAD4*, and *CDH2* (N-cadherin) genes (Figure 12B–E and Figure S3) as did the expression of Smad2/3, phospho-Smad2/3, and N-cadherin proteins (Figure 12G–L and Figure S3). Conversely, *CDH1* (E-cadherin) gene expression significantly increased in the 3-HBI and MO treatment groups (Figure 12A and Figure S3), as did E-cadherin protein expression (Figure 12F,L and Figure S3). These changes suggest reversal of EMT and restoration of epithelial characteristics in TNBC cells.

## 3. Discussion

Triple-negative breast cancer (TNBC) remains one of the most aggressive breast cancer subtypes due to its high recurrence rate, metastatic potential, and lack of targeted therapies [3,26]. In this study, we explored the potential of 3-hydroxy-β-ionone (3-HBI) identified from *Moringa oleifera* Lam. leaf extracts (MO), for its antitumor activity against TNBC using a dual approach that integrates in silico molecular docking and in vitro validation, with particular focus on the apoptosis and epithelial–mesenchymal transition (EMT) pathways mediated via TGF-β/Smad signaling.

3-HBI has been previously investigated for its anti-inflammatory properties [14,27] and anticancer effects in liver, lung, and head and neck cancers [17,18,19], and to the best of our knowledge, this is the first study evaluating its activity against TNBC. 3-HBI, beta-ionone compound having an (R)-hydroxy group at the 3-position [28]. This compound’s structural characteristics include its α, β-unsaturated carbonyl (enone) moiety at local at the local side chain: a conjugated double bond (C=C) adjacent to the carbonyl (C=O) at position 2 [28]. It may contribute to its ability to modulate signaling proteins and transcription factors involved in cancer progression [29]. In order to verify the activity of 3-HBI and guarantee consistency of results, we also assessed the effect of Moringa leaf extract on breast cancer cells in this study, which focuses on 3-HBI from MO. In our study, 3-HBI was identified as one of the components in Moringa leaf extract, but its actual concentration in the extract was not quantified. Previous reports, such as the study by Kouame Fulbert Oussou et al., showed that Moringa leaf infusions contain approximately 2700 µg/L of 3-HBI [30], which is considerably lower than the concentration used with the purchased compound. Moreover, the extract may contain other phytochemicals such as 3,4-Methyleneazelaic acid, (2S)-2-phenylmethoxybutane-1,4-diol, γ-Diosphenol, 2,2,4,4-Tetramethyl-6-(1-oxobutyl)-1,3,5-cyclohexanetrione, (2R)-2-phenylmethoxybutane-1,4-diol, and Tuberonic acid [14] that could exert synergistic or antagonistic effects, thereby contributing to the differences in biological activity.

Our findings indicate that 3-HBI may exert activity in TNBC, particular in the MDA-MB-231 cell line by targeting apoptosis and metastatic pathways, as supported by the in vitro and in silico data presented (Figure 4, Figure 5, Figure 6, Figure 7, Figure 8, Figure 9, Figure 10, Figure 11 and Figure 12). Further studies are required to confirm these effects in other TNBCs and in vivo.

3-HBI-induced breast cancer apoptosis is the beneficial outcome of this investigation (Figure 6 and Figure S1). In silico model results revealed favorable binding energies of 3-HBI with key apoptotic proteins, including Bax, Bcl-2, and caspase-3 (Figure 4 and Figure 5), indicating its potential to engage with the intrinsic apoptosis machinery. 3-HBI was predicted to interact with Leu within the BH3-binding groove of Bcl-2 (ΔG = −5.9 kcal/mol) via van der Waals forces, potentially impairing Bcl-2’s ability to sequester Bax and thereby promoting apoptosis [31,32]. This interaction is in agreement with the phenyl tetrahydroisoquinoline amide complex (ΔG = −10.3 kcal/mol), a known ligand previously re-docked to validate the docking protocol, which also binds to Bcl-2 at Leu. Consistently, treatment of MDA-MB-231 breast cancer cells with 3-HBI increased apoptotic cell death (Figure 6), accompanied by decreased Bcl-2 and significantly increased Bax expression (Figure 7). Furthermore, 3-HBI was predicted to interact with the α2–α5 region of Bax (Leu125, Pro130, Ile133, Met137; ΔG = −6.5 kcal/mol; Figure 4E), which is essential for its mitochondrial membrane insertion and cytotoxic function [32,33,34]. Although Bax was not subjected to re-docking validation, it exhibited the lowest binding free energy and formed interactions within the α2–α5 region, highlighting its functional relevance. Molecular dynamics simulation supported this interaction, showing Bax to be the most stable complex with 3-HBI, consistent with Bax upregulation and apoptosis induction in MDA-MB-231 cells (Figure 6 and Figure 7). In addition, 3-HBI interacted with Cys163 (Figure 4F), the catalytic cysteine crucial for proteolytic activity [35], which may alter enzymatic function. Notably, this interaction is consistent with the diverse P4 residues in peptide ligands (ΔG = −10.3 kcal/mol) observed in the caspase-3 crystal structure, which were confirmed through re-docking validation prior to docking with 3-HBI. This was supported by the observed increase in cleaved-caspase-3 in vitro (Figure 7). Moreover, 3-HBI was predicted to interact with caspase-9 at Arg177, Arg179, His237, Gln283, Cys285, Lys290, Val338, Ser339, Trp340, and Arg341, consistent with the binding mode of the benzoxycarbonyl-Val-Ala-Asp-fluoromethyl ketone inhibitor (ΔG = −7.5 kcal/mol) in the caspase-9 crystal structure, which was validated through re-docking prior to docking with 3-HBI. These results are consistent with the mechanism observed in previous studies: 3-HBI upregulated p53, Bax, cytochrome c, caspase-3, and caspase-9 and downregulated Bcl-2 in lung cancer A-549 cells [19], the same upregulated Bax, caspase-3, and downregulated Bcl-2 in squamous cell carcinoma SCC15 cells [17]. Nevertheless, these findings remain predictive and require further validation through co-immunoprecipitation, X-ray diffraction, and in vivo studies.

The main contribution of this work is that 3-HBI prevents TNBC metastasis by interfering with TGF-β/Smad-mediated EMT, which is a crucial mechanism for TNBC metastasis. Molecular docking and molecular dynamics simulation indicated that 3-HBI binds stably to Smad2 and Smad3, potentially preventing phosphorylation and nuclear translocation of the Smad2/3/4 complex (Figure 8 and Figure 9). Gene expression study revealed that *CHD1* (E-cadherin) was upregulated and *SMAD2*, *SMAD3*, *SMAD4*, and *CHD2* (N-cadherin) were downregulated in vitro, indicating epigenetic reprogramming that is detrimental to EMT. Additionally, protein analysis verified that Smad2, Smad3, and their phosphorylated versions (phospho-Smad2/Smad3) were suppressed. Furthermore, EMT indicators showed that TNBC migration and invasion were reduced as a result of decreased N-cadherin and increased E-cadherin (Figure 10, Figure 11 and Figure 12). The data suggest that 3-HBI could interfere with EMT in TNBC, particular in the MDA-MB-231. However, studies are needed to validate these effects in vivo and to assess their therapeutic potential. According to this study, 3-HBI is a promising dual-action option for MDA-MB-231 that can both reduce EMT and induce apoptosis. More in vivo validation, formulation development, and combination testing are necessary because this is the first evidence of its effectiveness in treating breast cancer, especially the most aggressive TNBC.

## 4. Materials and Methods

### 4.1. Cell Culture and Chemicals

MDA-MB-231 is the human TNBC cell line. MCF7 is a human ER-positive breast cancer cell line. CCD-1123Sk is a human fibroblast cell line. They were obtained from the American Type Cell Collection (ATCC, Manassas, VA, USA). Cells were cultured in Dulbecco’s modified eagle (DMEM) medium (Thermo Fisher Scientific, Waltham, MA, USA) with 10% fetal bovine serum (Thermo Fisher Scientific, Waltham, MA, USA) and 1% antibiotic-antimycotic (Thermo Fisher Scientific, Waltham, MA, USA) at 37 °C with 5% CO_2_. T47D is an ER, PR-positive breast cancer from CLS Cell Lines Service GmbH (Eppelheim, Germany), cultured in Roswell Park Memorial Institute (RPMI)-1640 medium (Thermo Fisher Scientific, Waltham, MA, USA) with 10% fetal bovine serum and 1% antibiotic-antimycotic. 3-hydroxy-β-ionone was from Santa Cruz Biotechnology (Dallas, TX, USA). 3-(4,5-Dimethylthiazol-2-yl)-2,5-Diphenyltetrazolium Bromide was from Thermo Fisher Scientific (Waltham, MA, USA). Muse™ Annexin V and Dead Cell reagent was from Merck Millipore (Burlington, MA, USA). Corning Matrigel Basement Membrane Matrix was from Corning, Inc. (Corning, NY, USA). Primary antibodies detecting Bax, Bcl-2, and caspase-3 were from Santa Cruz Biotechnology (Dallas, TX, USA), and anti-cleaved caspase-3 was from Affinity Biosciences (Cincinnati, OH, USA). β-actin antibody, rabbit anti-human IgG1 Fc secondary antibody conjugated with horseradish peroxidase (HRP), and mouse anti-human IgG1 Fc secondary antibody conjugated with HRP were from Thermo Fisher Scientific (Waltham, MA, USA). BlockPro^TM^ 1 Min Protein-Free Blocking Buffer was from Energenesis Biomedical (Taipei, Taiwan). Doxorubicin was purchased from Sigma-Aldrich, Inc., St. Louis, MO, USA.

### 4.2. Preparation of Moringa oleifera Lam. (MO) Extracts and Identification of Active Compounds

As stated in our earlier investigation, moringa leaves were isolated, and the active component was found [14]. In brief, Khaolaor Laboratories Co., Ltd. in Samutprakan, Thailand, provided the MO power. A 2 kg sample was sequentially extracted at room temperature using ethyl acetate (EtOAc) in three rounds (3 × 5 L). After the solvent evaporated, 128 g of crude EtOAc extract was produced. Using flash column chromatography, fractions and sub-fractions were extracted from the crude extract. Using LC-ESI-QTOF-MS/MS, an active molecule, 3-hydroxy-β-ionone (3-HBI), an apocarotenoid monoterpene, was discovered from the Moringa sub-fraction. Our present study used commercial 3-HBI with a purity of ≥90% from Santa Cruz Biotechnology (Dallas, TX, USA). MO extract was dissolved in 1:1 dimethyl sulfoxide (DMSO) and Tween 80, while 3-HBI was dissolved in 20% DMSO. The final percentage is 0.1% in experiments.

### 4.3. Cytotoxicity Assay

Breast cancer cell lines MDA-MB-231, T47D, MCF7, and fibroblast cell line CCD-1123Sk were cultured in 96-well plates at 1 × 10^4^ cells/well density and treated with MO extracts and 3-HBI for 24 h (0–1000 μg/mL in MDA-MB-231, 0–400 μg/mL in T47D, MCF7, and CCD-1123Sk). Then the cytotoxicity of MDA-MB-231, T47D, MCF7, and CCD-1123Sk was determined using the methyl thiazole tetrazolium (MTT) assay. A 0.5 mg/mL MTT solution was added to the plate, followed by incubation for 3 h at 37 °C, with 5% CO_2_ in a humidified incubator. Afterward, the formazan crystals were dissolved in 100 µL of DMSO, and absorbance was read at 590 nm with a microplate reader. (PerkinElmer Inc., Hopkinton, MA, USA).

Calculated inhibitory concentrations using the equation below, F is the percent of IC, H is the slope factor (Hill slope) from the graph and IC_50_ is 50% inhibitory concentration analyzed using GraphPad Prism 10.2.3 software.

$${IC}_{F}={\left(\frac{F}{100-F}\right)}^{\sqrt{H}}\cdot {\mathrm{IC}}_{50}$$

### 4.4. Colony Formation Assay

MDA-MB-231 cells were seeded into 12-well plates at 500 cells/well in DMEM completed medium and treated with doxorubicin, MO extract, and 3-HBI for 24 h. Then cells were washed and replaced with complete DMEM culture for 14 days. Cells were fixed with 10% neutral formalin and stained with 0.5% crystal violet to visualize colonies and photograph them. To dissolve crystal violet, 1 mL of 99.9% methanol was added to each well. The absorbance was measured at 570 nm using a microplate reader (PerkinElmer Inc., Hopkinton, MA, USA).

### 4.5. Migration Assay

In vitro scratch assay examined MDA-MB-231 migration. 2 × 10^5^ cells were plated into 24-well plates and cultured to 80–90% confluence. The wound area was scratched by the SPLScar™ Scratcher (SPL Life Sciences, Seoul, Republic of Korea). Cells were incubated with DMEM complete medium and treated with doxorubicin, MO extract, and 3-HBI for 24 h. The images were captured at 0, 6, 12, and 24 h using an inverted microscope (Zeiss Microscopy, Oberkochen, Germany). The percentage of wound closure was calculated using ImageJ software (Version 1.54j, Madison, WI, USA).

### 4.6. Transwell Invasion Assay

Next, 1 × 10^5^ of MDA-MB-231 with DMEM medium, doxorubicin, MO extract, and 3-HBI were seeded into the Matrigel-coated upper chambers of the Transwell (8 µm pore size) invasion assay system. The lower chamber contained DMEM with 10% FBS. After 24 h, invading cells were stained with 0.5% crystal violet and counted with a microscope. All experiments were repeated in triplicate with five fields of view counted on each membrane.

### 4.7. Reverse Transcription-Quantitative Real-Time PCR (RT-qPCR)

The total RNA was isolated by using a Trizol reagent (Invitrogen, Carlsbad, CA, USA) according to the manufacturer’s instructions. First-strand cDNA was synthesized using Ultrascript 2.0 synthesis kit (PCR Biosystems, London, UK). The thermal cycler program of cDNA synthesis involved incubation at 55 °C for 30 min followed by 95 °C for 10 min to inactivate reverse transcriptase. The synthesized cDNAs were amplified with specific primers using Qpcrbio sygreen mix separate-rox (PCR Biosystems, London, UK). RT-qPCR was conducted in three stages: an initial hold at 95 °C for 10 min; a PCR stage consisting of denaturation at 95 °C for 10 s, annealing at 58 °C for 10 s, and extension at 72 °C for 15 s, repeated for 40 cycles; and a final melting stage at 72 °C for 15 s followed by 95 °C for 15 s. Gene expression differences among groups were quantified based on CT values, normalized to the housekeeping gene GAPDH (ΔCT), and presented as fold-change over control (2^−ΔΔCT^) [36]. Real-time fluorescence was detected using the CFX96 Touch Real-Time PCR Detection System (Bio-Rad, Hercules, CA, USA). The oligonucleotide primers utilized in this study are listed in Table 3.

### 4.8. Western Blot Analysis

Cells were lysed in Radioimmunoprecipitation Assay (RIPA) buffer supplemented with 1× protease/phosphatase inhibitor cocktail for 5 min, ice cold and sonicated, and then centrifuged at 14,000× *g* for 10 min at 4 °C. Protein concentration was determined by the Bicinchoninic Acid (BCA) assay. Samples in different groups were separated into 12% Sodium Dodecyl Sulfate Polyacrylamide Gel Electrophoresis (SDS-PAGE) (100 V, 15 min, and 150 V, 1.30 h), and transferred onto Polyvinylidene Fluoride (PVDF) membranes (25 V, 1 A, 1 h) by Trans-Blot^®^Turbo transfer system (Bio-Rad, Hercules, CA, USA). The membranes were washed and blocked with BlockPro^TM^ 1 min protein-free blocking buffer for 1 min at room temperature. Then, the membranes are probed with primary antibodies (β-actin, Bcl2, Bax, caspase-3, cleaved caspase-3, Smad2/3, and phosphor-Smd2/3) overnight at 4 °C. The membranes were washed three times with Tris-Buffered Saline with Tween 20 (TBST) and incubated with secondary antibodies for 1 h at room temperature. Protein detection was performed by adding horseradish peroxidase chemiluminescent substrate. The intensity of each band was analyzed using Image Lab software, version 4.0.1.53407 (Bio-Rad, Hercules, CA, USA).

### 4.9. Molecular Docking

The molecular docking study used AutoDock Vina [37] to understand how 3-HBI binds to target proteins involved in apoptosis and the TGF-β/Smad pathway. Protein structures in PDB file format were downloaded from the RCSB Protein Data Bank and the AlphaFold Protein Structure Database, while the 3-HBI structure in SDF file format was downloaded from the PubChem chemical database (PubChem CID 11127505). Both protein and ligand were prepared by Discovery Studio 2021 using the chemistry tool and AutoDock Tools [38]. Before docking the proteins with 3-HBI, molecular docking protocols were validated by re-docking the co-crystallized ligand into the binding pocket, as indicated by a root mean square deviation (RMSD) value lower than 2 Å [39]. The docking was performed using a grid box with dimensions of 30 × 30 × 30 Å (x, y, z) and centered as shown in Table 4. AutoDock Vina was used for docking, and the results were visualized using BIOVIA Discovery Studio 2021.

### 4.10. Molecular Dynamics Simulation

The top three docking results with energies lower than −5 kcal/mol, related to apoptosis and the TGF-β/Smad pathway, were chosen for further study using molecular dynamics simulations. Molecular dynamics simulation of 3-HBI in complex with the proteins was carried out using the GROMACS 2022.4 software package on GPU accelerators [50]. The simulation protocol was based on a previously published method with slight modifications [51]. Protein structures were checked by BIOVIA discovery studio 2016 using the protein preparation tool, added missing amino acids by the SWISS-MODEL [52], and assigned to pH 7.4 by PROPKA [53]. The topology of the protein was constructed using the gmx_pdb2gmx tool and parameterized with the AMBER ff99SB force field. The ligand was parameterized with the General AMBER Force Field (GAFF). The charge distribution of 3-HBI was computed using the AM1-BCC method, facilitated by the acpype tool. The protein-ligand complexes were solvated in a TIP3P water model with a 12 Å buffer distance, which was prepared using the tleap tool (by AmberTools version 23.6). Na^+^ and Cl^−^ ions were added to neutralize the system to achieve a final ion concentration of 0.15 M. Energy minimization was conducted using the steepest descent method until the energy convergence of 10 kJ/mol/nm was reached. The system was equilibrated in two phases: initially under constant particle number, volume, and temperature (NVT), followed by continual particle number, pressure, and temperature (NPT) for 1000 ps in each phase. Molecular dynamics simulation productions were performed for 250 ns at 310 K and 1 bar, recording atomic coordinates every 10 ps. The resulting molecular dynamics simulation trajectories were analyzed using GROMACS tools, version 2022.4 and the RMSD was calculated with the gmx_rms utility. Hydrogen bonds were analyzed with gmx_hbond and visual molecular dynamics (VMD) software, version 1.9.3 applying a donor-acceptor distance cutoff of ≤3.5 Å and an angle cutoff of 20°. Protein backbone clustering was carried out using the GROMOS algorithm (on AMBER ff99SB, version 2006) with a 1.5 Å cutoff.

### 4.11. Statistical Analysis

Data are expressed as means ± SEM from three independent experiments. In the case of more than two groups, one-way ANOVA with post hoc multiple comparison corrections (Dunnett’s test) was conducted using GraphPad Prism 10.2.3 software. Statistical significance was defined as * *p* < 0.05, ** *p* < 0.01, *** *p* < 0.001, and **** *p* < 0.0001.

## 5. Conclusions

The findings indicate that 3-HBI reduces the growth of breast cancer by causing cell death, potentially by influencing the apoptosis pathway’s Bax, Bcl-2, and caspase-3. Furthermore, 3-HBI suppresses Smad2/3 in TGF-β/Smad signaling, which suppresses breast cancer metastasis. This study highlights 3-HBI as a promising candidate for further exploration in the development of novel anti-triple-negative breast cancer agents.

## Figures and Tables

**Table 1:** Molecular docking analysis of 3-HBI with target proteins in the apoptosis pathway.

Target Proteins	Estimate ΔG (kcal/mol)	Number of H-Bound	Amino Acid Interaction
**Bcl-2**	−5.9	0	Phe63, Tyr67, Asp70, Phe71, Met74, Lue96, Ala108, Phe11
**Bax**	−6.5	0	Gln28, Ile31, Gln32, Glu41, Ala42, Lue45, Ala46, Lue47, Lue125, Pro130, Ile133, Met137
**Caspase-9**	−5.2	3	Arg177, Arg179, His237, Gln283, Cys285, Lys290, Val338, Ser339, Trp340, Arg341
**Caspase-3**	−5.4	1	Thr62, Cys163, Tyr204, Ser205, Trp206, Arg207, Ser251, Phe256

**Table 2:** Molecular docking analysis of 3-HBI with target proteins in the TGF-β/Smad signaling pathway.

Target Proteins	Estimate ΔG (kcal/mol)	Number of H-Bound	Amino Acid Interaction
**Smad2**	−5.1	2	Glu270, Pro271, Ala272, Phe273, Trp274, Ala292, Ser293, Tyr340
**Smad3**	−5.4	0	Pro228, Tyr297, Gly300, Phe342, Asn344, Gly401, Pro402, Gln404, Trp405, Lys408
**Smad4**	−4.6	1	Asp52, Glu53, Ser56, Lys70, Cys71, Gln116, Tyr117, His132
**E-cadherin**	−4.0	1	Ile4, Pro5, Pro6, Ile7, Ser8, Leu21, Val22
**N-cadherin**	−4.6	1	His412, Asn417, Thr435, Pro437, Asn440, Gln467

**Table 3:** The oligonucleotide primers were utilized in this study.

Gene Code	Target Protein	Forward (5′ → 3′)	Reverse (5′ → 3′)
***BCL2***	B-cell lymphoma 2	GATGTGATGCCTCTGCGAAG	CATGCTGATGTCTCTGGAATCT
***BAX***	Bcl-2–associated X	GGTTGTCGCCCTTTTCTA	TGTTTCCCTGAGGTTTGC
***CASP3***	Caspase-3	GCTATTGTAGGCGGTTGT	TGTTTCCCTGAGGTTTGC
***SMAD2***	Suppressor of mothers against decapentaplegic 2	TGCTCTGAAATTTGGGGACTGA	GACGACCATCAAGAGACCTGG
***SMAD3***	Suppressor of mothers against decapentaplegic 3	ATCGTGAAGCGCCTGCTG	CATCCAGGGACCTGGGGA
***SMAD4***	Suppressor of mothers against decapentaplegic 4	GCCCGAGCCCAGGTTATC	ACAATGCTCAGACAGGCATCA
***CDH1***	E-cadherin	GAAATCACATCCTACACTGCCC	GTAGCAACTGGAGAACCATTGTC
***CDH2***	N-cadherin	AGAAGACCAGGACTATGACTTGAG	CACCACTACTTGAGGAATTAAGGG
***GAPDH***	Glyceraldehyde-3-phosphate dehydrogenase	ATGACATCAAGAAGGTGGTG	CATACCAGGAAATGAGCTTG

**Table 4:** Grid box of docking.

Target Protein	PDB ID/UniProt	Center x, y, z	References
**Bcl-2**	2W3L	x = 38.905, y = 26.949, z = −12.545	[40]
**Bax**	4S0O	x = 20.576, y = −0.177, z = 16.313	[41]
**Caspase-9**	1JXQ	x = 22.835, y = 39.722, z = −16.681	[42]
**Caspase-3**	3GJQ	x = 40.441, y = 33.843, z = 57.987	[43]
**Smad2**	6M64	x = −27.673, y = 5.506, z = −9.959	[44]
**Smad3**	1MK2	x = 21.095, y = −3.433, z = 0.421	[45]
**Smad4**	5MEY	x = −2.39, y = −16.454, z = 6.261	[46]
**E-cadherin**	4ZTE	x = 8.506, y = 13.076, z = 21.823	[47]
**N-cadherin**	A0A024RC42	x = −2.949, y = −2.211, z = −4.454	[48,49]

## Data Availability

The data presented in this study are available on request from the corresponding author.

## Supplementary Materials

The following supporting information can be downloaded at: https://www.mdpi.com/article/10.3390/ijms26188771/s1.

## Abbreviation

The following abbreviations are used in this manuscript:

TNBC	Triple-negative breast cancer
EMT	Epithelial-to-mesenchymal transition
3-HBI	3-hydroxy-β-ionone
MO	*Moringa oleifera* Lam. leaf extract
Dox	Doxorubicin
Smad	Suppressor of mothers against decapentaplegic
