# Supplementary material for: 3-Hydroxy-β-ionone Suppresses Breast Cancer Progression by Inducing Apoptosis and Blocking EMT Through the TGF-β/Smad Signaling Pathway
Source: Int J Mol Sci. 2025 Sep 9;26(18):8771. doi: 10.3390/ijms26188771 (PMC12469291; doi:10.3390/ijms26188771)
Supplement: Supplementary file 1 [file ijms-26-08771-s001.zip › ijms-3809522-supplementary.pdf]

Supplementary Material

3-Hydroxy-β-Ionone suppresses breast cancer progression by inducing apoptosis and blocking EMT through the TGF-β/Smad signaling pathway

Pornsuda Sutana [1], Thitiya Luetragoon [1,2], Watunyoo Buakaew [1,3], Krai Daotak [1], Nungruthai Nilsri [1], Yordhathai Thongsri [1], Pachuen Potup [1], Catherine Léon [4] and Kanchana Usuwanthim [1,*]

*Correspondence: kanchanau@nu.ac.th

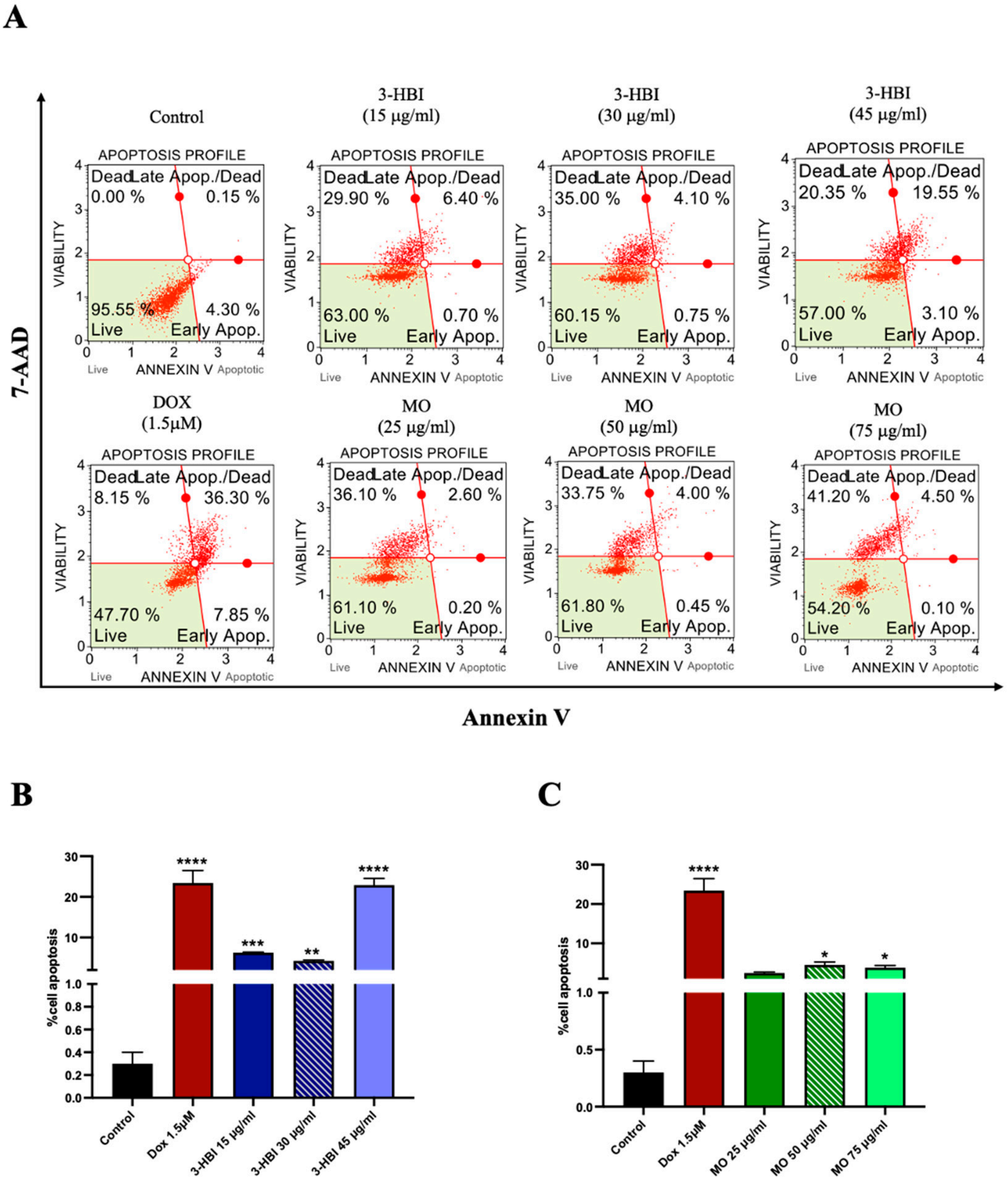

Figure S1. The apoptosis of the T47D cell line was analyzed using a Muse cell analyzer. (A) T47D apoptosis profiles. Bar graphs of T47D apoptosis, (B) 3-HBI treatment groups (C) MO treatment groups. Data are presented as means ± SEM. * $p \leq 0.05$, ** $p \leq 0.01$, *** $p \leq 0.001$, and **** $p \leq 0.0001$ compared to control. Control: untreated T47D; 3-HBI: 3-hydroxy-β-ionone; MO: *Moringa oleifera* Lam.

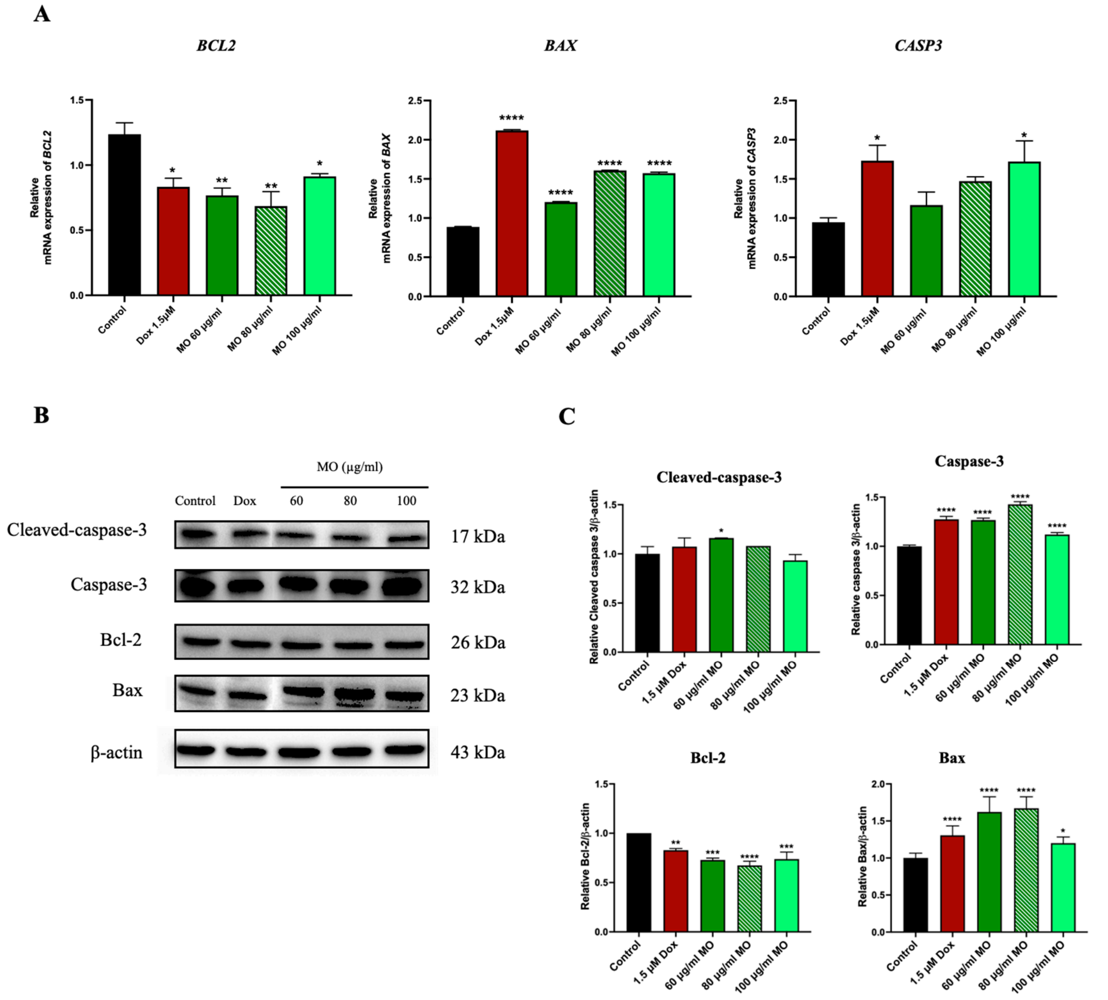

Figure S2. Effect of MO on apoptotic gene and protein expression (A) The expression of *BCL2*, *BAX*, and *CASP3* genes of the 3-HBI treatment groups. (B) Apoptosis proteins expression and (C) Bar graphs of apoptosis proteins. Data are presented as means ± SEM. * $p \leq 0.05$, ** $p \leq 0.01$, *** $p \leq 0.001$, and **** $p \leq$ 0.0001 compared to control. Control: untreated MDA-MB-231; MO: *Moringa oleifera* Lam.

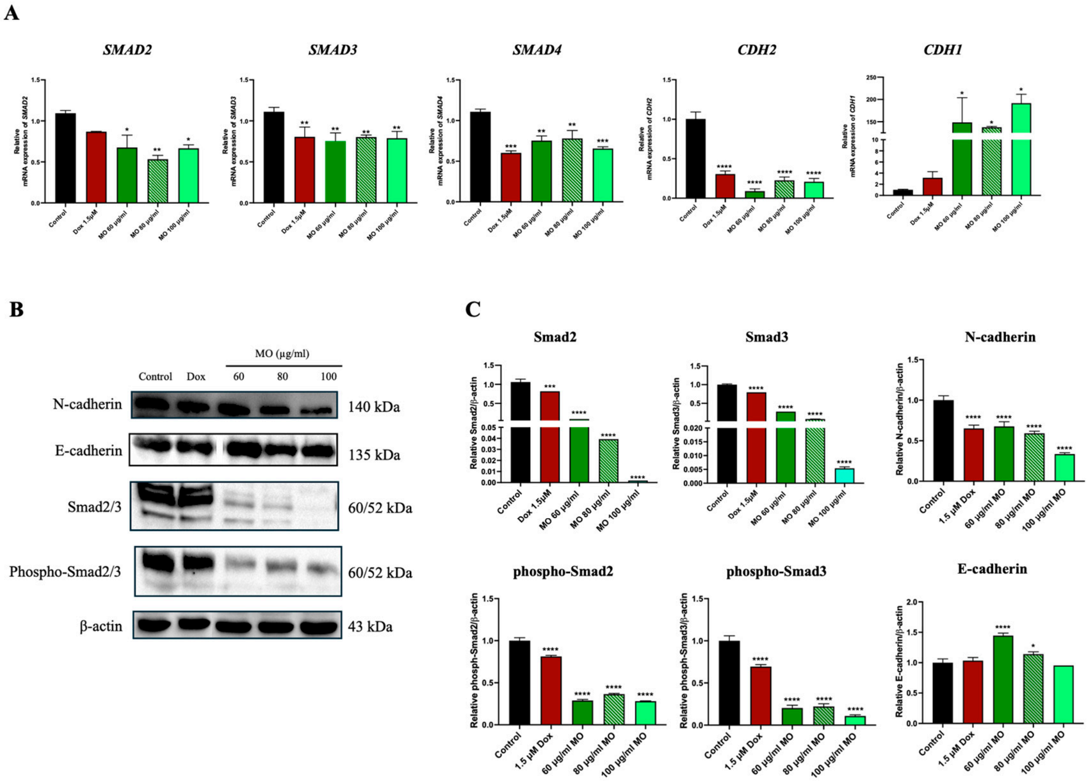

Figure S3. Effect of MO on TGF-β/Smad pathway-related gene and protein expression. (A) The expression of SMAD2, SMAD3, SMAD4, CDH2 and CDH1 genes in MO treatment groups. (B) N-cadherin, E-cadherin, Smad2/3 and phospho-Smad2/3 expression and (C) Bar graphs of proteins. Data are presented as means ± SEM. * $p \leq 0.05$, ** $p \leq 0.01$, *** $p \leq 0.001$, and **** $p \leq 0.0001$ compared to control. Control: untreated MDA-MB-231; MO: *Moringa oleifera* Lam.
